# Association between the chronic use of gastric acid suppressants and high‐risk colorectal polyps

**DOI:** 10.1002/jgh3.12503

**Published:** 2021-01-29

**Authors:** Yasutoshi Shiratori, Takashi Ikeya, Naoki Ishii, Kazuki Yamamoto, Tetsuro Honda, Kenkei Hasatani, Naohiro Yoshida, Tsutomu Nishida, Tetsuya Sumiyoshi, Shu Kiyotoki, Masahiro Arai, Ryota Niikura

**Affiliations:** ^1^ Department of Gastroenterology St. Luke's International Hospital Tokyo Japan; ^2^ Department of Gastroenterology Tokyo Shinagawa Hospital Tokyo Japan; ^3^ Department of Gastroenterology Nagasaki Harbor Medical Center Nagasaki Japan; ^4^ Department of Gastroenterology Fukui Prefectural Hospital Fukui Japan; ^5^ Department of Gastroenterology Ishikawa Prefectural Central Hospital Kanazawa Japan; ^6^ Department of Gastroenterology Toyonaka Municipal Hospital Osaka Japan; ^7^ Department of Gastroenterology Tonan Hospital Sapporo Japan; ^8^ Department of Gastroenterology Shuto General Hospital Yanai Japan; ^9^ Department of Gastroenterology Nerima Hikarigaoka Hospital Tokyo Japan; ^10^ Department of Gastroenterology, Graduate school of medicine The University of Tokyo Tokyo Japan; ^11^ Clinical Research Promotion Center The University of Tokyo Hospital Tokyo Japan

**Keywords:** colonoscopy, colorectal neoplasms, histamine‐2 receptor antagonists, polyps, proton pump inhibitors

## Abstract

**Background and Aim:**

Although gastric acid suppressants such as proton pump inhibitors (PPIs) and histamine‐2 receptor antagonists (H2RAs) are considered safe, the consequences of hypochlorhydria and hypergastrinemia caused by chronic use are unclear. This study aimed to investigate the association between the chronic use of gastric acid suppressants and high‐risk colorectal polyps, focusing on polyp size.

**Methods:**

A population‐based, nested case–control study was conducted using data from the Japanese Diagnosis Procedure Combination database between 2014 and 2019. Cumulative PPI or H2RA use prior to polypectomy was evaluated during the study period. Endoscopic polypectomy was categorized as polypectomy <2 cm, polypectomy ≥2 cm, and endoscopic submucosal dissection. Baseline characteristics were compared between the high‐risk (≥2 cm polyps or polyps treated by endoscopic submucosal dissection) and low‐risk (<2 cm polyps) endoscopic polypectomy groups. We calculated adjusted odds ratios (ORs) using multivariable logistic regression analysis.

**Results:**

Of 27 694 patients who underwent endoscopic polypectomy, 2518 were treated with PPIs or H2RAs for >1 year prior to polypectomy. After adjusting for age, gender, and other confounders, a higher prevalence of high‐risk colorectal polyps was noted with PPI (OR: 2.67; 95% confidence interval: 2.37–3.01) and H2RA (OR: 1.86; 95% confidence interval: 1.52–2.26) use. Longer PPI or H2RA use was associated with increased risks of high‐risk colorectal polyps (*P* for trend <0.001). The highest OR (3.17) was observed among patients who received PPIs for ≥3 years.

**Conclusion:**

Chronic use of PPIs and H2RAs may be associated with high‐risk colorectal polyps. Requirements for long‐term gastric acid suppressant use should be reevaluated.

## Graphical abstract

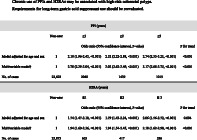

## Introduction

Gastric acid suppressants, such as proton pump inhibitors (PPIs) and histamine‐2 receptor antagonists (H2RAs), are effective against diseases such as gastroesophageal reflux disease and gastric ulcers. These are the most commonly prescribed medications globally.^1^, ^2^ Most patients are chronically prescribed gastric acid suppressant therapies, and this chronic administration has been linked to side effects such as gastrointestinal infections; diarrhea; carcinoma, particularly that of the upper gastrointestinal tract; and mortality.^1^, ^3^, ^4^, ^5^, ^6^, ^7^, ^8^, ^9^, ^10^ A recent report revealed that hypergastrinemia due to PPIs might be correlated with sustained gastric atrophy following *Helicobacter pylori* eradication^11^ and could be associated with the incidence of gastrointestinal cancers.^12^, ^13^, ^14^, ^15^, ^16^ In addition, patients with Zollinger‐Ellison syndrome have highly proliferative colorectal epithelial tumors.^17^ However, owing to insufficient evidence regarding the correlation between PPIs and colorectal cancer, it is unclear whether the use of PPI and H2RA is correlated with the malignant potential of colorectal tumors. Several observational studies have reported a potential relationship between PPIs and colorectal malignancy.^13^, ^14^ However, other studies have reported no such association.^1^, ^2^, ^18^, ^19^, ^20^ In addition, most prior studies were population‐based studies on the prevalence of colorectal cancer that lacked sufficient PPI exposure periods and adequate descriptions of colonoscopy findings such as polyp size. Colorectal polyps are known to have a high rate of carcinogenesis depending on their size; the rate of invasion of the submucosal layer by a carcinoma is higher at polyp sizes >20 mm.^21^ Therefore, we aimed to investigate whether chronic use of PPIs or H2RAs increases the odds of high‐risk colorectal polyps among Japanese patients.

## Methods

### *Design, participants, and data sources*

This retrospective, nested case–control study was based on the Japanese Diagnosis Procedure Combination database of personal academic groups, including St. Luke's International Hospital, Tokyo University, Nagasaki Harbor Medical Center, Fukui Prefectural Hospital, Ishikawa Prefectural Central Hospital, Toyonaka Municipal Hospital, Tonan Hospital, Shuto General Hospital, and Nerima Hikarigaoka Hospital. The database includes the following data: patient age and gender, diagnosis and comorbidities coded with the International Classification of Disease and Related Health Problems Tenth Revision codes (ICD‐10), and procedures and drugs coded with the original Japanese codes. Personal information was deleted from the data collected for analysis from each participating facility. This study was approved by the Institutional Review Board of the University of Tokyo Hospital (no. 2019161NI). Informed consent was waived because of the retrospective nature of the study.

### *Endoscopic polypectomy*

From the database, we extracted the data of adult patients (≥20 years) who underwent endoscopic polypectomy, including those who were prescribed oral PPIs and H2RAs before the procedure, from January 2014 to March 2019. For patients who underwent two or more polypectomies during the study period, only data related to the initial polypectomy were included, while data from the second polypectomy and any subsequent polypectomies were excluded. Endoscopic polypectomy procedures were categorized as follows: (i) polypectomy of tumors <2 cm, (ii) polypectomy of tumors ≥2 cm, and (iii) endoscopic submucosal dissection (ESD). Tumor size was measured by the endoscopist, nurse, or technician after polypectomy. Cold polypectomy or endoscopic mucosal resection was performed for tumors <2 cm, but only endoscopic mucosal resection was performed for tumors ≥2 cm. Details of the ICD‐10 codes are shown in Table S1.

### *Medication*

We assessed the use of PPIs, H2RAs, low‐dose aspirin, nonsteroidal anti‐inflammatory drugs (NSAIDs), cyclooxygenase 2 (COX‐2) inhibitors, and metformin. PPIs included vonoprazan, esomeprazole, rabeprazole, lansoprazole, and omeprazole. H2RAs included famotidine, ranitidine, cimetidine, roxatidine, nizatidine, and lafutidine. We defined exposure as continuous PPI or H2RA use for >1 year prior to undergoing polypectomy upon consideration of polyp progression. Patients taking PPIs or H2RAs for <1 year were excluded from the analyses. Other drugs were considered confounders if they were administered at the time of polypectomy. Details of the drug codes are shown in Table S2.

### *Outcomes and confounders*

The primary outcome was the prevalence of high‐risk colorectal polyps (≥2 cm polyps or polyps treated by ESD) among patients who underwent polypectomy based on the use of PPIs and H2RAs. We evaluated data on age, gender, comorbidities, medication, and type of endoscopic polypectomy. The following 13 diseases were evaluated as comorbidities: cerebrovascular disease, arterial thrombosis, pulmonary disease, chronic heart failure, ischemic heart disease, gastrointestinal ulcers, liver disease, chronic kidney disease, deep vein thrombosis, malignant tumor with no metastasis, malignant tumor with metastasis, hypertension, and diabetes. Details of the ICD‐10 codes of the aforementioned comorbidities are shown in Table S3.

### *Statistical analysis*

Primary statistical analyses were performed using Stata ver. 16 (StataCorp LP, College Station, TX, USA). We compared the baseline characteristics between two groups, namely, the high‐risk (polypectomy of polyps ≥2 cm or polyps treated by ESD) and low‐risk (polypectomy of polyps <2 cm) endoscopic polypectomy groups, using Student's *t*‐test or chi‐square test for continuous or categorical variables. We examined the risk factors of the primary outcome, that is, the prevalence of high‐risk polyps. Factors with a *P*‐value of <0.05 in the univariate logistic regression model, as well as age and gender, were included in the multivariable logistic regression model. Multivariable logistic regression analysis was performed to adjust for potential confounders and to calculate odds ratios (ORs) and 95% confidence intervals (CIs) for high‐risk colorectal polyps among patients receiving PPIs or H2RAs with reference to the values obtained among those not receiving PPIs or H2RAs. As a subanalysis, we evaluated patients based on the duration of PPI and H2RA use (≥1, ≥2, and ≥3 years). We additionally evaluated the interaction between PPIs and H2RAs. We divided the patients into groups according to the duration of PPI use as (PPI ≥1 year *H2RA), (PPI ≥2 years *H2RA), and (PPI ≥3 years *H2RA). We then reperformed the multivariable logistic regression analysis.

Missing data for gender and age were imputed using the fitted values from general linear models for comorbidities using SAS ver. 9.4 (SAS Institute, Cary, NC, USA). A two‐tailed *P*‐value <0.05 was considered indicative of statistical significance.

## Results

Of the 27 694 patients who underwent polypectomy from January 2014 to March 2019 (excluding patients' second polypectomy and any subsequent polypectomies, if applicable), 2518 received PPIs or H2RAs for >1 year prior to polypectomy. Of the 2518 patients who received gastric acid suppressants, 1915, 470, and 133 received PPIs, H2RAs, and both PPIs and H2RAs, respectively. Among the entire patient cohort, 10 949 underwent polypectomy for tumors <2 cm, 15 466 underwent polypectomy for tumors ≥2 cm, and 1279 underwent ESD.

Table 1 presents the distribution of patients' characteristics and risk factors for colorectal polyps according to the category of the endoscopic polypectomy procedure employed. The high‐risk group (patients with ≥2 cm polyps or polyps treated by ESD) displayed greater prevalence of cerebrovascular disease, arterial thrombosis, chronic heart disease, ischemic heart disease, gastrointestinal ulcer, malignant tumor with no metastasis, hypertension, diabetes, PPI use, and COX‐2 inhibitor use than did the low‐risk group (patients with <2 cm polyps).

**Table 1:** Characteristics of 27 694 subjects according to the history of endoscopic polypectomy

	High‐risk colorectal polyps		Low‐risk colorectal polyps
	Polypectomy ≥2 cm (*n* = 15 466)	Endoscopic submucosal dissection (*n* = 1279)	Polypectomy <2 cm (*n* = 10 949)
Mean age	68.1	67.5	67.4
≥70	8141 (52)	607 (48)	5468 (50)
<70	7325 (48)	672 (52)	5481 (50)
Gender‐no. subjects (%)			
Male	7887 (51)	696 (54)	5255 (48)
Female	5104 (33)	583 (46)	3722 (34)
No data	2475 (16)	0	1972 (18)
Medical conditions‐no. subjects (%)			
Cerebrovascular disease	436 (3)	59 (5)	165 (2)
Arterial thrombosis	64 (0.4)	11 (1)	24 (0.2)
Pulmonary disease	293 (2)	29 (2)	186 (2)
Chronic heart failure	586 (4)	51 (4)	267 (2)
Ischemic heart disease	145 (1)	18 (1)	42 (0.4)
Gastrointestinal ulcer	1306 (8)	208 (16)	733 (7)
Liver disease	403 (3)	41 (3)	276 (3)
Chronic kidney disease	275 (2)	15 (1)	123 (1)
Deep vein thrombosis	84 (1)	10 (1)	39 (0.3)
Malignant tumor no metastasis	863 (6)	133 (10)	434 (4)
Malignant tumor with metastasis	204 (1)	15 (1)	113 (1)
Hypertension	1321 (9)	229 (18)	545 (5)
Diabetes	1315 (9)	218 (17)	803 (7)
Medication‐no. subjects (%)			
PPI	1404 (9)	83 (7)	563 (5)
H2RA	381 (3)	32 (3)	196 (2)
Aspirin	752 (5)	92 (7)	695 (6)
Nonsteroidal anti‐inflammatory drugs	2218 (14)	158 (12)	1528 (14)
Cyclooxygenase‐2 inhibitor	309 (2)	36 (3)	436 (4)
Metformin	273 (2)	32 (3)	199 (2)

H2RA, histamine‐2 receptor antagonist; PPI, proton pump inhibitor.

Table 2 presents the logistic regression analysis findings for the prevalence of high‐risk colorectal polyps. Multivariable logistic regression analysis revealed that PPI and H2RA use was associated with the prevalence of high‐risk colorectal polyps (both *P* < 0.01).

**Table 2:** Results of logistic regression models for the prevalence of high‐risk colorectal polyps

	Crude OR (95% CI)	*P*‐value	Adjusted OR (95% CI)	*P*‐value
PPI	1.74 (1.58–1.92)	<0.001	2.67 (2.37–3.01)	<0.001
H2RA	1.37 (1.15–1.62)	<0.001	1.86 (1.52–2.26)	<0.001
Age				
<70 (reference)	1		1	
≥70	1.10 (1.04–1.16)	0.009	1.01 (0.94–1.08)	0.92
Gender				
Female (reference)	1		1	
Male	0.92 (0.86–0.98)	0.008	1.00 (0.93–1.07)	0.95
Medical conditions				
Cerebrovascular disease	3.06 (2.59–3.60)	<0.001	1.58 (1.31–1.90)	<0.001
Arterial thrombosis	2.81 (1.86–4.24)	<0.001	1.57 (0.91–2.25)	0.08
Pulmonary disease	2.11 (1.77–2.53)	<0.001	1.15 (0.95–1.41)	0.16
Chronic heart failure	2.92 (2.54–3.36)	<0.001	1.49 (1.26–1.76)	<0.001
Ischemic heart disease	3.73 (2.76–5.05)	<0.001	2.33 (1.66–3.28)	<0.001
Gastrointestinal ulcer	2.27 (2.08–2.48)	<0.001	1.57 (1.43–1.74)	<0.001
Liver disease	1.98 (1.71–2.29)	<0.001	1.48 (1.25–1.75)	<0.001
Chronic kidney disease	3.10 (2.52–3.81)	<0.001	1.76 (1.39–2.22)	<0.001
Deep vein thrombosis	2.63 (1.85–3.75)	<0.001	1.34 (0.94–1.78)	0.13
Malignant tumor with no metastasis	2.44 (2.19–2.72)	<0.001	1.36 (1.20–1.54)	<0.001
Malignant tumor with metastasis	2.46 (1.97–3.08)	<0.001	1.71 (1.33–2.20)	<0.001
Hypertension	2.83 (2.58–3.10)	<0.001	1.85 (1.66–2.06)	<0.001
Diabetes	2.10 (1.93–2.29)	<0.001	1.07 (0.96–1.18)	0.19
Medication				
Aspirin	0.78 (0.71–0.87)	<0.001	0.80 (0.73–0.90)	0.01
Nonsteroidal anti‐inflammatory drugs	0.96 (0.88–1.02)	0.58		
Cyclooxygenase‐2 inhibitor	0.77 (0.55–0.94)	<0.001	0.72 (0.53–0.92)	<0.001
Metformin	1.00 (0.84–1.20)	0.98		

Age, gender, and factors with a *P*‐value of <0.05 in the univariate model (PPI, H2RA, cerebrovascular disease, arterial thrombosis, pulmonary disease, chronic heart failure, ischemic heart disease, gastrointestinal ulcer, liver disease, chronic kidney disease, malignant tumor with no metastasis, malignant tumor with metastasis, hypertension, diabetes, aspirin, and cyclooxygenase‐2 inhibitor) were included in the multivariable logistic regression model.

ESD, endoscopic submucosal dissection; H2RA, histamine‐2 receptor antagonist; PPI, proton pump inhibitor; OR, odds ratio.

There was an apparent trend toward an increased risk of high‐risk colorectal polyps with increasing duration of PPI (*P* for trend: 0.01) and H2RA (*P* for trend: 0.01) exposure (Table 3).

**Table 3:** Subgroup analysis using logistic regression models for the association between the period of medication use and the prevalence of high‐risk colorectal polyps

	PPI (years)				
	Nonuser	≥1	≥2	≥3	
	Odds ratio (95% confidence interval, *P*‐value)				*P* for trend
Model adjusted for age and gender	1	2.18 (1.96–2.43, <0.001)	2.52 (2.22–2.88, <0.001)	2.74 (2.35–3.21, <0.001)	<0.001
Multivariable model^†^	1	2.70 (2.39–3.04, <0.001)	3.02 (2.62–3.49, <0.001)	3.17 (2.68–3.75, <0.001)	<0.001
No. of cases	22 602	2048	1439	1019	
	Nonuser	≧1	≧2	≧ 3	
	Odds ratio (95% confidence interval, *P*‐value)				*P* for trend
Model adjusted for age and gender	1	1.74 (1.47–2.10, <0.001)	1.79 (1.43–2.24, <0.001)	2.06 (1.56–2.72, <0.001)	0.004
Multivariable model^†^	1	1.94 (1.60–2.36, <0.001)	1.94 (1.54–2.45, <0.001)	2.18 (1.62–2.90, <0.001)	<0.001
No. of cases	25 875	603	417	286	

^†^ Data in the multivariable model were adjusted for age, gender, cerebrovascular disease, arterial thrombosis, ischemic heart disease, chronic heart failure, pulmonary disease, gastrointestinal ulcer, liver disease, chronic kidney disease, deep vein thrombosis, malignant tumor without metastasis, malignant tumor with metastasis, hypertension, diabetic mellites, aspirin, NSAIDs, COX‐2 inhibitor, and metformin.

H2RA, histamine‐2 receptor antagonist; PPI: proton pump inhibitor.

Table 4 presents the association between PPI use and high‐risk colorectal polyps according to H2RA use. No interaction was observed between PPI and H2RA use (*P* = 0.39).

**Table 4:** Subgroup analysis using multivariable logistic regression models for the association between PPI and H2RA in the group with high‐risk colorectal polyps

	H2RA user		H2RA nonuser			Interaction between administration of PPI and H2RA
	Odds ratio (95% confidence interval)			Odds ratio (95% confidence interval)		
	High‐risk polyps			High‐risk polyps		
PPI			PPI			
Nonuser			Nonuser			
Current user	1	*P*‐value	Current user	1	*P*‐value	*P*‐val
≥1 year	2.79 (2.43–3.14)	<0.001	≥1 year	2.61 (2.31–2.96)	<0.001	0.24
≥2 years	3.16 (2.69–3.65)	<0.001	≥2 years	2.97 (2.56–3.45)	<0.001	0.34
≥3 years	3.33 (2.77–3.94)	<0.001	≥3 years	3.14 (2.64–3.73)	<0.001	0.39

Data in the multivariable model were adjusted for age, gender, cerebrovascular disease, arterial thrombosis, ischemic heart disease, chronic heart failure, pulmonary disease, gastrointestinal ulcer, liver disease, chronic kidney disease, deep vein thrombosis, malignant tumor without metastasis, malignant tumor with metastasis, hypertension, diabetic mellites, aspirin, NSAIDs, COX‐2 inhibitor, and metformin.

COX‐2, cyclooxygenase‐2; H2RA, histamine‐2 receptor antagonist; NSAIDs, nonsteroidal anti‐inflammatory drugs; PPI, proton pump inhibitor.

## Discussion

The long‐term clinical implications of hypochlorhydria and hypergastrinemia induced by gastric acid suppressant use are not completely clear. This study demonstrated an association between the use of gastric acid suppressants and high‐risk colorectal polyps. This finding is consistent with the results of two other population‐based studies that examined the association between PPIs and colorectal cancer risk.^13^, ^14^

Here, a multivariable regression analysis revealed higher ORs for high‐risk colorectal polyps with PPI (OR: 2.67) and H2RA (OR: 1.86) exposure than for low‐risk colorectal polyps with no PPI or H2RA exposure. Therefore, PPI and H2RA exposure may be an independent risk factor for high‐risk colorectal polyps. A higher cumulative period of gastric acid suppressant use was associated with an increased risk of high‐risk colorectal polyps. The highest OR was observed among patients who received PPIs for ≥3 years. In the present study, we evaluated interactions between PPI administration and H2RA use and found no such interaction, and the ORs were approximately the same regardless of H2RA use. We considered that effect modifications did not occur based on the administration of H2RA and that each medication was an independent risk factor. H2RAs reduce hypogastric acid production to a lesser extent than do PPIs, which may explain why H2RAs were associated with slightly lower odds of high‐risk colorectal polyp occurrence than were PPIs.

In this study, the prevalence of high‐risk colorectal polyps among patients who underwent polypectomy was as high as 60% (16 746/27 694). However, there was a potential selection bias related to the database we used, which included selective patients who did not represent all those receiving colonoscopy in the general population. Potential bias or confounders were as follows. First, we mainly focused on inpatients from nine high‐volume hospitals. Second, patients requiring gastric acid suppressants because of comorbidities show a higher risk of malignancy relative to that in the regional database. Third, large polyps may cause constipation or other diffuse gastrointestinal symptoms and, consequently, require PPIs.

We defined long‐term administration as administration for at least 1 year, which is similar to or longer than the definitions used in other studies on PPI use and the risk of gastrointestinal cancer (defined as current use). A population‐based cohort study examining the association between PPIs and colorectal cancer in South Korea defined PPI exposure as administration for 60 days.^14^ In addition to the relatively short duration of exposure, that study assessed the prevalence of colorectal cancer using a population‐based database, but it was not clear whether the entire population underwent colonoscopy within the study period. In a study published in Taiwan, the mean duration of exposure to PPIs was 75 days.^13^ However, it is not logical that a few months of exposure would increase the risk of a disease with a long latency period, such as colorectal cancer. Prior studies that showed no association between PPIs and colorectal cancer had defined current PPI administration using 1‐year^15^ and 2‐year^20^ durations. Although it is difficult to evaluate the correct index date, a mean follow‐up of 1–3 years might not be sufficient to investigate gastric acid suppressants as a risk factor for colorectal cancer. This study assessed the risk of colorectal polyps based on polyp size from colorectal polypectomy events rather than based on a diagnosis of colorectal cancer; we found that, while not all polyps >2 cm led to colorectal cancer, they did indicate an increased risk of colorectal cancer. The mean (standard deviation) duration of PPI and H2RA use in the current study were 1136 (11.6) and 1101 (19.9) days, respectively. We consider this to be an adequate duration for PPIs and H2RAs to affect colorectal polyp growth. It is important to note that PPIs are not available over the counter in Japan as they are in other countries such as the United States and Sweden.^3^, ^20^ Aspirin is known to be useful for the prevention of colorectal cancer.^22^ However, after adjusting for medications such as aspirin, COX‐2 inhibitors, and NSAIDs, our multivariable logistic regression analysis revealed a twofold increased risk of high‐risk colorectal polyps among patients receiving long‐term gastric acid suppressants compared to that among patients not receiving gastric acid suppressants.

ESD is generally performed for early‐stage colorectal cancer tumors ≥2 cm in diameter, neuroendocrine tumors, or early cancers with fibrosis. No size‐based relationship between colorectal polyp grade and gastric acid suppressants has been previously reported. Previous animal studies have demonstrated that the proliferation of colorectal cancer is stimulated by progastrin^23^, ^24^ and that hypergastrinemia might promote colonic adenoma progression.^25^ These studies showed that patients receiving gastric acid suppressants were assumed to have high‐risk colorectal polyps.

A strength of our study is that we examined high‐risk colorectal polyps categorized according to size from a population‐based database including information on polypectomy procedures. We also studied H2RAs in addition to PPIs. Long‐term PPI and H2RA use were associated with high‐risk colorectal polyps. In addition, the trend of high‐risk colorectal polyps was associated with the duration of PPI and H2RA use. Although the switch from PPI use to H2RA use was sometimes made in Japan, chronic H2RA use was also observed to be associated with high‐risk colorectal polyps.

This study has several limitations. First, this was a retrospective study. Second, although we performed logistic regression analyses, unmeasured confounders may exist. Third, the database did not include information about the endoscopists or the location, morphology, or histopathology of the colorectal polyps. Finally, data from the Japanese Diagnosis Procedure Combination database regarding endoscopic polypectomy have not been validated yet. Further chart reviews regarding the diagnostic sensitivity and specificity are required. This study evaluated the prevalence of high‐risk polyps based on PPI and H2RA use. Proof of causality of PPIs or H2RAs for high‐risk polyps requires further prospective cohort studies to examine temporality (Hill's criteria).^26^

In conclusion, long‐term PPI and H2RA use may be associated with high‐risk colorectal polyps. Therefore, requirements for long‐term gastric acid suppressant use should be reevaluated.

## Supporting information


**Table S1** Receipt codes for endoscopic polypectomy
**Table S2**. Receipt codes for medications
**Table S3**. ICD‐10 codes for comorbidities
