# Supplementary material for: Association between the chronic use of gastric acid suppressants and high‐risk colorectal polyps
Source: JGH Open. 2021 Jan 29;5(3):371–6. doi: 10.1002/jgh3.12503 (PMC7936615; doi:10.1002/jgh3.12503)
Supplement: Supplementary file 1 — Table S1 Receipt codes for endoscopic polypectomy Table S2. Receipt codes for medications Table S3. ICD‐10 codes for comorbidities [file JGH3-5-371-s001.docx]

**SUPPORTING INFORMATION**

**Online Resource 1. Receipt codes for endoscopic polypectomy**

| Polypectomy (<2 cm) |
| --- |
| K7211 |
| Polypectomy (≥2 cm) |
| K7212 |
| Endoscopic submucosal dissection |
| K7214 |

**Online Resource 2. Receipt codes for medications**

| Combination agents of pylori eradication | 610462048, 610462049, 622029101, 622289101, 622289201, 622289301, 622485401, 622485501, 622485601 |
| --- | --- |
| Amoxicillin | 620006829, 620008584, 620009117, 621073901, 622745400, 616130040, 616130132, 620006919, 616130295, 620007025 |
| Clarithromycin | 616140105, 620003926, 620003927, 620003928, 620003929, 620003930, 620003931, 620003932, 620003933, 620003934, 620003935, 620003939, 620006670, 620008013, 621736701, 621742103, 621752901, 622079401, 622659101, 622746200, 616140102 |
| Metronidazole | 620007057 |
| Proton Pump Inhibitors | 610412202, 610412203, 610443068, 610443069, 610443070, 610443071, 610462010, 610462011, 612320549, 612320550, 620001983, 620001984, 620001985, 620001986, 620002694, 620002695, 620002743, 620002744, 620002749, 620002750, 620002871, 620002872, 620003914, 620004087, 620004088, 620005581, 620005583, 620005584, 620005585, 620007127, 620007128, 620009451, 620009452, 621622303, 621622403, 621630104, 621630502, 621630601, 621630701, 621673701, 621673801, 621680901, 621681001, 621681401, 621693101, 621693201, 621743701, 621743801, 621780301, 621780303, 621780401, 621780403, 621794301, 621796301, 621796401, 621919001, 621919101, 621977902, 621983103, 621997201, 621997301, 621999501, 621999601, 621999901, 622000001, 622001201, 622001301, 622002201, 622002301, 622005301, 622005401, 622007301, 622007401, 622011201, 622011301, 622012901, 622013001, 622015801, 622015901, 622016201, 622016301, 622020602, 622020702, 622021401, 622021501, 622023101, 622023201, 622023301, 622023401, 622025601, 622025701, 622026001, 622026101, 622031601, 622031701, 622032801, 622032901, 622034601, 622034701, 622035501, 622035601, 622040101, 622040201, 622060201, 622060301, 622077801, 622080701, 622080801, 622089501, 622089601, 622118601, 622118701, 622402601, 622469801, 622469901, 622505501, 622505601, 622617000, 622617100,622617200, 622624801,622624901 |
| Potassium Competitive Acid Blockers | 622404401, 622404501 |
| Nonsteroidal Anti-Inflammatory Drugs | 610406382, 610406383, 610406384, 610406387, 610406388, 610406402, 610422322, 610433119, 610443079, 610443080,  610454052, 610463033, 610463034, 610463037, 610463150,  611140098, 611140138, 611140139, 611140236, 611140237,  611140322, 611140323, 611140395, 611140431, 611140435,  611140828, 611140844, 611140845, 611140846, 611140847,  620002043, 620002057, 620002431, 620002432, 620002516,  620002531, 620002537, 620002646, 620002647, 620003153,  620003154, 620003523, 620003524, 620003624, 620004494,  620004626, 620004857, 620004858, 620004916, 620004917,  620006095, 620006174, 620006848, 620006849, 620006859,  620007059, 620007068, 620007095, 620007096, 620007098,  620007099, 620007100, 620007129, 620007150, 620007151,  620007152, 620007153, 620008114, 620008115, 620008117,  620008118, 620008119, 620008120, 620008121, 620008122, 620008123, 620008124, 620008125, 620008126, 620008127,  620008128, 620008129, 620008130, 620008131, 620008132,  620008133, 620008135, 620008136, 620008137, 620008138,  620008139, 620008140, 620008141, 620008142, 620008143,  620008144, 620008145, 620008146, 620008147, 620008148,  620008149, 620008150, 620008151, 620008625, 620008628,  620008632, 620008646, 620008780, 620079303, 620079305,  620079311, 620079315, 620079325, 620079338, 620079345,  620081301, 620088902, 620090601, 620094401, 620097508,  620097815, 620098401, 620098501, 620098702, 620098801,  620098902, 620099003, 620099101, 620099201, 620099301,  620099501, 620099601, 620099701, 620100001, 620100501,  620100602, 620100702, 620100901, 621212601, 621215101,  621215401, 621215602, 621392002, 621466002, 621466202,  621466401, 621466601, 621534501, 621623201, 621634301,  621635802, 621640201, 621640501, 621808201, 621837703,  621837803, 621936001, 621981502, 622011102, 622012401,  622014601, 622022501, 622034902, 622051201, 622058201,  622062601, 622066701, 622314000, 622325600 |
| Aspirin | 610443053, 611140017, 611140798, 611140849, 611140850,  620000065, 620000484, 620001952, 620004280, 620007816,  620008577, 620009301, 620072734, 621362001, 621374801,  621374901, 621375001, 621391201, 621419201, 621419401,  621675501, 621676502, 622258001 |
| Statin | 610443013, 610443014, 610454084, 610454085, 610462015,  610462016, 610470012, 610470013, 610470014, 612180263,  612180264, 612180265, 620000052, 620000053, 620000103, 620000104, 620000105, 620000106, 620000107, 620000108, 620000159, 620000160, 620000176, 620000422, 620000423, 620002477, 620002478, 620002736, 620002798, 620002799, 620002800, 620004038, 620008053, 620008054, 620008055, 620008056, 620009322, 620009323, 620009324, 620009325, 621521301, 621521401, 621523101, 621523201, 621524102, 621524402, 621525701, 621525801, 621528602, 621528702,  621528801, 621528901, 621529001, 621529101, 621531001, 621531101, 621531703, 621532501, 621532601, 621532902,  621533002, 621533101, 621533201, 621533501, 621533601, 621533801, 621533901, 621534003, 621534101, 621534204,  621534301, 621623603, 621635202, 621639001, 621639101, 621639701, 621639801, 621643301, 621643401, 621643501  621643601, 621675101, 621694001, 621752501, 621934801, 621934901, 621935001, 621948701, 621948801, 621955003,  621964101, 621964201, 621964301, 621964401, 621964501, 621964601, 621981403, 622015101, 622015201, 622015301, 622052801, 622055602, 622071601, 622075801, 622075901, 622076401, 622076501, 622098401, 622098501, 622099101,  622099201, 622102502, 622107601, 622107701, 622110401, 622110501, 622116802, 622116902, 622126901, 622127001,  622128201, 622128301, 622136401, 622139600, 622143801, 622143901, 622152001, 622152101, 622161801, 622161901,  622165601, 622165701, 622167601, 622167701, 622169902, 622170002, 622170101, 622170201, 622180602, 622180702,  622186601, 622186701, 622187601, 622187701, 622204801, 622204901, 622217101, 622217201, 622239201, 622239301,  622241301, 622241401, 622244801, 622244901, 622252001, 622252101, 622268001, 622268101, 622268201, 622269101,  622269201, 622270001, 622270101, 622271801, 622271901, 622273101, 622273201, 622273301, 622274901, 622275001,  622275101, 622276301, 622276401, 622276501, 622280201, 622280301, 622280401, 622280501, 6222806,01, 622280701, 622280801, 622282201, 622282301, 622283701, 622283801, 622285001, 622285101, 622286201, 622286301, 622286401, 622287601, 622289501, 622289601, 622291801, 622291901, 622292001, 622292301, 622292401, 622292501, 622293301, 622293401, 622294301, 622294401, 622294501, 622296001, 622296101, 622296201, 622297101, 622297201, 622298001, 622298101, 622298201, 622299001, 622299101, 622302401, 622302501, 622302801, 622302901, 622304601, 622304701, 622304801, 622304901, 622315400, 622315500, 622321900, 622342801, 622347401, 622359101, 622360101, 622362701, 622365801, 622372401, 622387601, 622392501, 622406901, 622419701, 622419801, 622419901, 622421601, 622421701, 622421801, 622426201, 622427701, 622427801, 622431401, 622434301, 622434401, 622441101, 622441201, 622457701, 622457801, 622457901, 622464901, 622465001, 622465101, 622475000, 622475100, 622512001, 622512101, 622512201, 622522101, 622522201, 622522301, 622524501, 622524601, 622524701, 622528901, 622529001, 622537301, 622537401, 622568601, 622571801, 622571901, 622572801, 622572901, 622575201, 622575301, 622575401, 622575501, 622575601, 622575701, 622577901, 622578001, 622578101, 622578201, 622578401, 622578501, 622578601, 622578701, 622578801, 622581601, 622581701, 622581801, 622581901, 622582001, 622582101, 622582501, 622582601, 622582701, 622582801, 622584201, 622584701, 622584801, 622586001, 622586101, 622586201, 622586301, 622588801, 622588901, 622589001, 622589101, 622590101, 622590201, 622590301, 622590401, 622591701, 622591801, 622591901, 622592001, 622592601, 622592701, 622592901, 622593001, 622593101, 622593201, 622595301, 622595401, 622598301, 622598401, 622598501, 622598601, 622599201, 622599301, 622599401, 622599501, 622600301, 622600601, 622600701, 622600801, 622600901, 622601201, 622601301, 622601401, 622601501, 622604001, 622604101, 622604201, 622605001, 622605101, 622605201, 622605301, 622605401, 622606601, 622606701, 622615600, 622615700, 622615800, 622615900, 622640801, 622640901, 622644801, 622644901, 622660001, 622660101, 622665901, 622666001, 622666101, 622676701, 622676801, 622691000, 622691800, 622692400, 622692500, 622692600, 622692700 |
| Fibrate | 610407028, 610422262, 610422263, 610422264, 610422265,  610422276, 612180028, 612180029, 612180106, 620002123,  620008508, 620338317, 620339201, 620339401, 620339501,  620340201, 620340603, 620340901, 620341001, 620341301,  621254601, 622026702, 622039501, 622039601, 622090701,  622090801, 622096102, 622096801, 622096901, 622223601,  622573101, 622590501, 622590601 |
| Other lipid lowering | 610432003, 610462007, 610463087, 612180004, 612180140,  612180141, 612180292, 620002508, 620003669, 620004459,  620004868, 620005785, 620005920, 620006115, 620006870,  620008631, 620346008, 620346018, 620346023, 620346029, 620346101, 622198801, 622516701, 622516801, 622516901, 622584201, 622584701, 622676701, 622676801 |
| Metformin | 620004480, 620005570, 621676001, 621974701, 621986301,  621986401, 622242501, 622412701, 622417101, 622417201,  622421101, 622421201, 622421901, 622422001, 622424401,  622424501, 622427201, 622427301, 622432601, 622432701, 622436301, 622438401, 622438501, 622448601, 622450301, 622450401, 622466601, 622517101, 622654401, 622654501 |

**Online Resource 3. ICD10 codes for comorbidities**

| Comorbidities | ICD-10 code |
| --- | --- |
| Arterial thrombosis | I740-I749 |
| Carotid disease | I652, I720 |
| Cerebrovascular disease | G450-G469, H340, I600-639, I64, I650-I699 |
| Chronic heart failure | I099, I110, I130, I132, I255, I420, I425-I439, I500-I509, P290 |
| Chronic kidney disease < stage 5 | I120, I131, N032-N037, N052-N057, N180-N189, N19, N250, Z490-Z492, Z940, Z992 |
| Chronic kidney disease stage 5 | N185 |
| DM without complications | E100, E101, E106, E108-E111, E116, E118-E121, E126, E128-E131, E136, E138-E141, E146, E148, E149 |
| DM with complications | E102-E105, E107, E112-E117, E122-E125, E132-E135, E137, E142-E145, E147 |
| Deep vein thrombosis | I800-I809, I820-I829 |
| Hypertension | I10, I110-I159 |
| Dyslipidemia | E780-E785 |
| Ischemic heart disease | I210-I229, I252 |
| Unstable angina disease | I200-I209 |
| Liver disorder (mild) | B180-B189, K700-K703, K709, K713-K715, K717, K730-K749, K760, K762-K764, K768-K769, Z944 |
| Liver disorder (severe) | I850, I859, I864, I982, K704, K711, K721, K729, K765-K767 |
| Malignancy without metastasis | C000-C009, C01, C020-C69, C07, C080-C119, C12, C130-C189, C19, C20, C210-C229, C23, C240-C329, C33, C340-C349, C37, C380-C519, C52, C530-C549, C55, C56, C570-C570, C58, C600- C609, C61, C620-C639, C64, C65, C66, C670-C729, C73, C740-C769, C810-C969, C97 |
| Malignancy with metastasis | C770-C809 |
| Pulmonary embolism | I260-I269 |
| Peripheral vascular disease | I700-I719, I731, I738, I739, I771, I790, I792, K551, K558, K559, Z958, Z959 |
| Pulmonary disease | I278, I279, J40, J410—J419, J42, J430-J459, J46, J47, J60, J61, J620-J639, J64, J65, J660-J679, J684, J701, J703 |
| Transient ischemic attack | G459 |
| Peptic ulcer disease | K250-K289 |
